# Evaluating RAG Configurations for Clinical Information Extraction from EHR Notes: Aged Care Case Study

**DOI:** 10.1007/s41666-026-00254-8

**Published:** 2026-09-14

**Authors:** Dinithi Vithanage, Quang Vinh Duong, Chao Deng, Rajendra Acharya, Zhanzhong Gu, Ting Song, Ping Yu

**Affiliations:** 1 School of Computing and Information Technology, University of Wollongong, Wollongong, Australia; 2 School of Medical, Indigenous and Health Sciences, University of Wollongong, Wollongong, Australia; 3 School of Mathematics, Physics and Computing, University of Southern Queensland, Darling Heights, Australia; 4 School of Electrical and Data Engineering, University of Technology Sydney, Broadway, Australia

**Keywords:** Natural language processing, Large language models, Retrieval augmented generation, Clinical information extraction, Named entity recognition

## Abstract

Clinical information extraction from unstructured electronic health records is important for supporting clinical decision making and healthcare research. However, large language models can struggle to accurately extract domain-specific information without effective adaptation. Retrieval-augmented generation offers a promising approach, but the optimal configuration for clinical information extraction remains unclear. This study evaluated different retrieval-augmented generation configurations for extracting frailty index items, malnutrition risk factors, and agitation in dementia from clinical notes collected from 40 Australian residential aged care facilities. We compared general and biomedical embedding models, LangChain and LlamaIndex retrieval frameworks, top-k values of 1, 5, 10, 15, and 20, and one to five few-shot examples using Llama-3.1-8B-Instruct. Performance was evaluated using accuracy, precision, recall, and F1 score. The best results were achieved using LangChain with the BAAI/bge-m3 embedding model, three-shot prompting, and a top-k value of five. This configuration achieved F1 scores of 97.08% for frailty index extraction, 97.14% for malnutrition risk factor extraction, and 90.52% for agitation in dementia extraction. These findings provide empirical evidence on how retrieval, embedding, and prompting choices influence clinical information extraction performance. The study offers practical guidance for researchers and healthcare informatics practitioners seeking to optimise retrieval-augmented large language model systems for extracting clinically meaningful information from unstructured aged care records.

**Supplementary Information:**

The online version contains supplementary material available at https://doi.org/10.1007/s41666-026-00254-8.

## Introduction

The volume of data stored in electronic health records (EHRs) has increased dramatically in recent years, with the majority consisting of unstructured content such as clinical notes [1–3]. These notes often contain rich, observational insights that are crucial for clinical decision-making and care planning [1]. However, their free-text format poses significant challenges for computational processing, as they are often written in inconsistent styles and include diverse terminology, abbreviations, acronyms, and narrative structures [3]. To make this information accessible and actionable, it must first be converted into a structured format [1, 2]. This requires advanced information extraction (IE) methods capable of identifying key clinical entities, such as symptoms, diagnoses, risk factors, and treatments, from narrative text [1].

Recent advances in natural language processing (NLP), particularly the development of generative large language models (LLMs) like GPT-4, Claude, Gemini, and Llama, have introduced powerful tools for clinical IE [1, 3–5]. Although LLMs are exposed to broad biomedical literature during pre-training, this general knowledge does not capture the highly localised, institutional characteristics of residential aged care documentation [1, 5]. Nursing notes in aged care facilities are heavily abbreviated, telegraphic, and shaped by facility-specific conventions that are unlikely to be well-represented in standard pre-training corpora [1, 3, 5]. Furthermore, unlike relatively structured clinical documents such as discharge summaries or radiology reports, aged care nursing notes are written under significant time pressure by staff with variable documentation training, resulting in non-standard, fragmented text that presents a substantially harder IE subproblem. In this context, retrieval-augmented generation (RAG) addresses not merely a factual knowledge gap, but also a local and institutional knowledge gap that parametric knowledge alone cannot reliably bridge. Retrieval-augmented generation (RAG) is an architecture that enhances this adaptation [1, 6, 7]. By supplementing pretrained knowledge with task-specific context, RAG enhances LLMs by integrating external information retrieval into an LLM’s generation process, boosting accuracy and relevance. It improves clinical IE from sources like EHRs [8], making LLMs more effective for structuring unstructured clinical notes [1, 6].

However, the practical application of RAG with generative LLMs in real-world clinical settings remains limited. Most existing research focuses on mainstream datasets or controlled environments, often overlooking the variability and complexity of real-world clinical notes [1]. Importantly, RAG behaves differently in the clinical domain compared to mainstream data and tasks, as clinical text is highly specialized, noisy, and context-dependent, and errors in interpretation can have significant real-world consequences [1, 5]. Consequently, identifying and optimising appropriate RAG configurations is critical for reliable clinical IE. Current studies fall short in identifying the most effective RAG configuration for clinical tasks, leaving a gap in understanding how to optimize LLMs with RAG for use in real-world healthcare workflows. To address this gap, this study aims to determine the most effective RAG configurations for clinical IE through a series of experiments guided by the hypotheses outlined in Table 1. This study contributes to improving the reliability, scalability, and clinical relevance of LLM-driven clinical IE systems and ultimately supports their integration into everyday clinical workflows.

**Table 1:** Research hypotheses to investigate performance disparities across different RAG configurations

Research hypotheses	
1	Embedding models have varied performance in clinical information extraction in an RAG architecture.
2	RAG framework implementation in LangChain or LlamaIndex affects information retrieval performance
3	Increasing the number of few-shot examples beyond three in prompts improves the performance of clinical IE in an RAG architecture.
4	Varying the number of top-k retrieved documents impacts the performance of clinical information extraction in an RAG architecture.

## Methodology

### Ethics Approval

The Human Research Ethics Committee of the University of Wollongong approved the study (Ethics Number 2019/159).

### Dataset Description

Deidentified clinical notes were collected from 40 residential aged care facilities (equivalent to nursing homes in the US) belonging to a management group in New South Wales, Australia, between 2019 and 2021.

### Clinical Information Extraction Task Selection

This study focuses on three clinical IE tasks: (1) identification of frailty index items, (2) extraction of malnutrition risk factors, and (3) detection of agitation in dementia. The dataset for the frailty index was originally annotated following the procedures detailed in Zhu et al. [9] by the large research group. The annotating process involved associating each nursing note with one or more of the 36 frailty index items defined by Clegg et al. [10]. The annotated dataset for malnutrition was based on the work of Alkhalaf et al. [11], and the annotated dataset for agitation in dementia was derived from the studies by Zhu et al. [12, 13]. A complete list of labels used in the frailty index, malnutrition risk factors, and agitation in dementia tasks is provided in Supplementary Table 1.

### RAG Pipeline Development

**Fig. 1:**
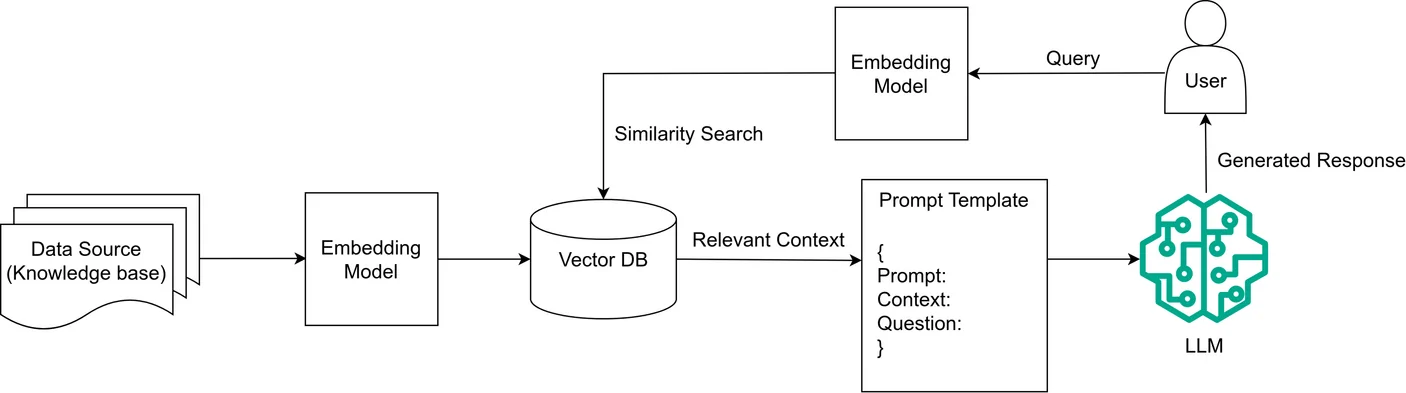
Baseline RAG pipeline

A baseline RAG pipeline (see Fig. 1) includes the following key stages: constructing a domain-specific knowledge base, generating embeddings for relevant data, storing these embeddings in a vector database, implementing efficient retrieval mechanisms with top-k retrieval parameters, designing few-shot prompts, applying an LLM for entity extraction, and evaluating information extraction performance.

#### Knowledge Base Construction

A knowledge file for the frailty index was developed using the annotated dataset described in Sect. 2.3. This file was formatted as a CSV with two columns: the first column contained relevant definitions, symptoms, and commonly occurring keywords extracted from the clinical notes, while the second column mapped the phrases in the clinical notes to the corresponding items in three IE tasks: frailty index, malnutrition risk factors, and agitation in dementia.

Each RAG knowledge file was formatted as a CSV file with two columns. The first column contained a clinical note. The second column contained the relevant clinical keywords or phrases (e.g., frailty indicator items, malnutrition risk factors, or agitation-related symptoms) extracted from the clinical note. Table 2 shows an example of the RAG knowledge file for the frailty index items. Similarly, the RAG file for the malnutrition risk factors task was drawn from the study by Alkhalaf et al. [6], and the file for the agitation in dementia task was adapted from the work of Vithanage et al. [1]. These studies were all conducted on the same data sets detailed in Sect. 2.3.

**Table 2:** A snippet of a RAG knowledge file for frailty index items

Clinical notes	Frailty Index
Difficulties an individual may have in executing activities. It refers to a restriction in a person’s ability to perform tasks or actions that are typically expected of them due to physical, mental, or sensory impairments.	Activity limitation
Mobility problems. Refer to difficulties in moving from one place to another, including walking, climbing stairs, or navigating uneven surfaces.	Mobility problems
Transfer problems involve challenges in moving from one position to another, such as from a bed to a chair, sitting to standing, or transferring between different surfaces.	Transfer problems

#### Embedding Generation

The knowledge sources were converted into dense vector representations (embeddings) suitable for semantic search. To evaluate how different embedding strategies impact retrieval effectiveness in clinical IE, we selected a range of embedding models inspired by prior research [14, 15]. These include general-purpose models such as Alibaba-NLP/gte-large-en-v1.5 [16], BAAI/bge-large-en-v1.5, and BAAI/bge-m3 [17], as well as domain-specific biomedical models like dmislab/biobert-v1.1 [17], emilyalsentzer/Bio_ClinicalBERT [18, 19] and MedCPT [20].

#### Vector Database Storage

The generated embeddings were stored in a vector database to facilitate efficient retrieval. In this experiment, we used FAISS [21] for its efficient index structure for fast retrieval, scalability, and flexibility in handling high-dimensional vector data [22, 23].

#### RAG Frameworks and Top-k Retrieval Configurations

After storing the knowledge embeddings, we retrieved relevant information from the vector database. We compared two widely used RAG frameworks: LangChain and LlamaIndex. For each framework, we tested top-k retrieval with k values of 1, 5, 10, 15, and 20 to determine how the number of retrieved documents impacts clinical information extraction performance.

#### Few-Shot Prompting

Vithanage et al. [1] recommend using RAG combined with few-shot learning as an effective approach for clinical IE. Based on this, we adopted few-shot prompting as the primary strategy within our RAG framework, with the system prompt manually designed based on domain knowledge and prior studies [1, 5]. We compared multiple configurations using 1-shot, 2-shot, 3-shot, 4-shot, and 5-shot examples to assess the impact of different numbers of demonstrations on model performance. The few-shot demonstration examples were manually selected to represent different clinical scenarios and kept fixed across all experiments, rather than being randomly sampled or dynamically selected during inference. The same fixed prompt configuration was applied consistently across all experiments to ensure fairness and reproducibility. The prompt examples used in our study are provided in Supplementary Table 2.

#### Large Language Model Application

To extract clinical entities, the retrieved documents were combined with the original query and a few-shot prompt examples, then fed to an LLM. We selected the Llama-3.1-8B-Instruct model based on the following considerations: (1) its superior performance among open-source models and favourable evaluations at the time of experimentation; (2) the practical availability of compatible GPU resources; (3) its availability for deployment on local servers, offering greater convenience and control; (4) its compliance with Australian health data privacy regulations; and (5) the model’s extensive ecosystem, which includes fine-tuned variants such as Alpaca, Baize, Koala, and Vicuna [1].

### Performance Evaluation

The information retrieval performance was evaluated using test datasets specific to each clinical IE task: frailty index (*n* = 100), malnutrition risk factors (*n* = 300), and agitation in dementia (*n* = 200). The model-generated outputs were compared against annotated ground truth data, as detailed in Sect. 3.3. To assess the performance, we calculated accuracy, F1 score, precision, and recall, in line with the evaluation strategies recommended by previous studies [1]. The evaluation was performed on the Gadi supercomputer provided by the Australian National Computational Infrastructure (NCI) [24], which features 48 CPUs, four NVIDIA Tesla V100 GPUs (each with 32 GB of memory), and 382 GB of system memory.

### Statistical Analysis

We first assessed the distribution of each performance metric using the Shapiro–Wilk test for normality, following prior recommendations [25]. As the metrics (accuracy, precision, recall, and F1 score) did not follow a normal distribution, we applied a logit transformation to stabilize variance and approximate normality, in line with prior studies [26, 27]. We further assessed the assumption of homogeneity of variances using Levene’s test [28]. When this assumption was satisfied, a one-way ANOVA was conducted for comparisons involving three or more independent groups. For two-group comparisons, independent-samples t-tests were applied. These statistical methods align with those recommended in previous research [29–31]. The null hypothesis in each test assumed that there was no significant difference in performance between groups, while the alternative hypothesis suggested a significant difference. A p-value less than 0.05 was considered statistically significant, leading to the rejection of the null hypothesis. To control for false positives arising from multiple comparisons, we applied the Bonferroni correction, as recommended in prior studies [32].

## Results and Discussions

This section presents the study’s findings in response to each research hypothesis outlined in Table 1. The findings are followed by a discussion of their implications in the context of these research hypotheses. The experimental results are detailed in Supplementary Table 3.

### Testing Hypothesis 1: Embedding Models Have Varied Performance in Clinical Information Extraction in an RAG Architecture

#### Results

To test Hypothesis 1, different embedding models were evaluated within both the standard RAG architecture and a MedRAG [33] enhanced retrieval architecture using a three-shot learning configuration, as recommended by Vithanage et al. [5]. The MedRAG configuration incorporated medical-domain retrieval enhancements, including hybrid retrieval and medical-aware query expansion, to assess whether domain-specific retrieval strategies further improved clinical information extraction performance [33].

Although the differences in performance between embedding models within the standard RAG and MedRAG frameworks were not statistically significant, consistent variations in performance were observed among the evaluated embedding models. Among all models, the MedCPT embedding model achieved the highest overall performance across both RAG and MedRAG (see Table 3). Within the standard RAG, MedCPT attained an accuracy of 94.00% (± 0.03%), F1-score of 97.02% (± 0.02%), precision of 97.04% (± 0.02%), and recall of 96.99% (± 0.02%). Under the MedRAG, performance improved further, achieving an accuracy of 90.18% (± 0.02%), F1-score of 97.14% (± 0.01%), precision of 97.29% (± 0.01%), and recall of 97.00% (± 0.02%).

**Table 3:** Performance comparison between different embedding models

RAG	Method	Accuracy	F1-score	Precision	Recall
Standard RAG	Alibaba-NLP/gte-large-en-v1.5	88.21% (± 0.01%)	88.95% (± 0.01%)	89.96% (± 0.01%)	89.93% (± 0.01%)
	BAAI/bge-largeen-v1.5	90.41% (± 0.03%)	92.24% (± 0.01%)	92.24% (± 0.01%)	92.24% (± 0.02%)
	**MedCPT**	**94.00% (± 0.03%)**	**97.02% (± 0.02%)**	**97.04%(± 0.02%)**	**97.00%(± 0.02%)**
	dmis-lab/biobert-v1.1	92.71% (± 0.03%)	94.25% (± 0.02%)	94.33% (± 0.02%)	94.16% (± 0.02%)
	emilyalsentzer/Bio ClinicalBERT	89.00% (± 0.01%)	90.42% (± 0.01%)	90.52% (± 0.02%)	90.32% (± 0.01%)
	BAAI/bge-m3	93.21% (± 0.01%)	95.01% (± 0.01%)	96.74%(± 0.02%)	95.00%(± 0.02%)
MedRAG	Alibaba-NLP/gte-large-en-v1.5	87.91% (± 0.01%)	91.50% (± 0.05%)	92.44% (± 0.02%)	90.55% (± 0.01%)
	BAAI/bge-largeen-v1.5	91.44% (± 0.01%)	94.86% (± 0.07%)	95.66% (± 0.05%)	94.07% (± 0.02%)
	**MedCPT**	**94.18% (± 0.02%)**	**97.14% (± 0.01%)**	**97.29% (± 0.01%)**	**97.00% (± 0.02%)**
	dmis-lab/biobert-v1.1	88.47% (± 0.05%)	95.57% (± 0.02%)	96.31% (± 0.05%)	94.82% (± 0.02%)
	emilyalsentzer/Bio ClinicalBERT	88.81% (± 0.02%)	91.28% (± 0.02%)	90.50% (± 0.04%)	92.05% (± 0.02%)
	BAAI/bge-m3	93.21% (± 0.01%)	95.01% (± 0.01%)	96.74%(± 0.02%)	95.00%(± 0.02%)

In contrast, the Alibaba-NLP/gte-large-en-v1.5 model consistently demonstrated the lowest performance across both retrieval architectures. Within the standard RAG framework, it achieved an accuracy of 88.21% (± 0.01%), F1-score of 88.94% (± 0.01%), precision of 89.96% (± 0.01%), and recall of 89.93% (± 0.01%). Similarly, within the MedRAG framework, the model continued to underperform, recording an accuracy of 87.91% (± 0.01%), F1-score of 91.50% (± 0.05%), precision of 92.44% (± 0.02%), and recall of 90.55% (± 0.01%).

#### Discussion

Among the evaluated embedding models, MedCPT consistently achieved the highest performance across both RAG and MedRAG architectures, demonstrating superior results in accuracy, F1-score, precision, and recall for clinical information extraction tasks. However, although MedCPT outperformed all other models across configurations, the observed differences between embedding models were not statistically significant, indicating that performance variations should be interpreted as trends rather than definitive superiority. Notably, models specifically trained on biomedical corpora, such as BioBERT and ClinicalBERT, did not achieve the expected leading performance despite their domain-specific pretraining. A possible explanation is that these models were primarily trained on formal biomedical literature and structured clinical datasets (e.g., PubMed and MIMIC-III ICU notes) [18], which differ substantially from the characteristics of aged care clinical documentation used in this study. These source datasets typically contain standardized medical terminology and well-structured clinical narratives, which may not generalize effectively to more heterogeneous real-world aged care notes. In contrast, the aged care clinical notes in our dataset include substantial informal documentation, such as colloquial expressions, non-standard abbreviations, shorthand notations, and narrative-style observations. These linguistic characteristics differ markedly from formal biomedical text and may reduce the effectiveness of models trained exclusively on structured clinical corpora. Interestingly, MedCPT demonstrated stronger and more consistent performance, which may be attributed to its training on more diverse and broadly distributed corpora that better capture varied linguistic patterns, including informal and heterogeneous text expressions. This broader exposure may enhance its ability to generalize to real-world clinical documentation settings such as aged care environments.

### Testing Hypothesis 2: RAG Framework Implementation in LangChain or LlamaIndex Affects Information Retrieval Performance

#### Results

**Fig. 2:**
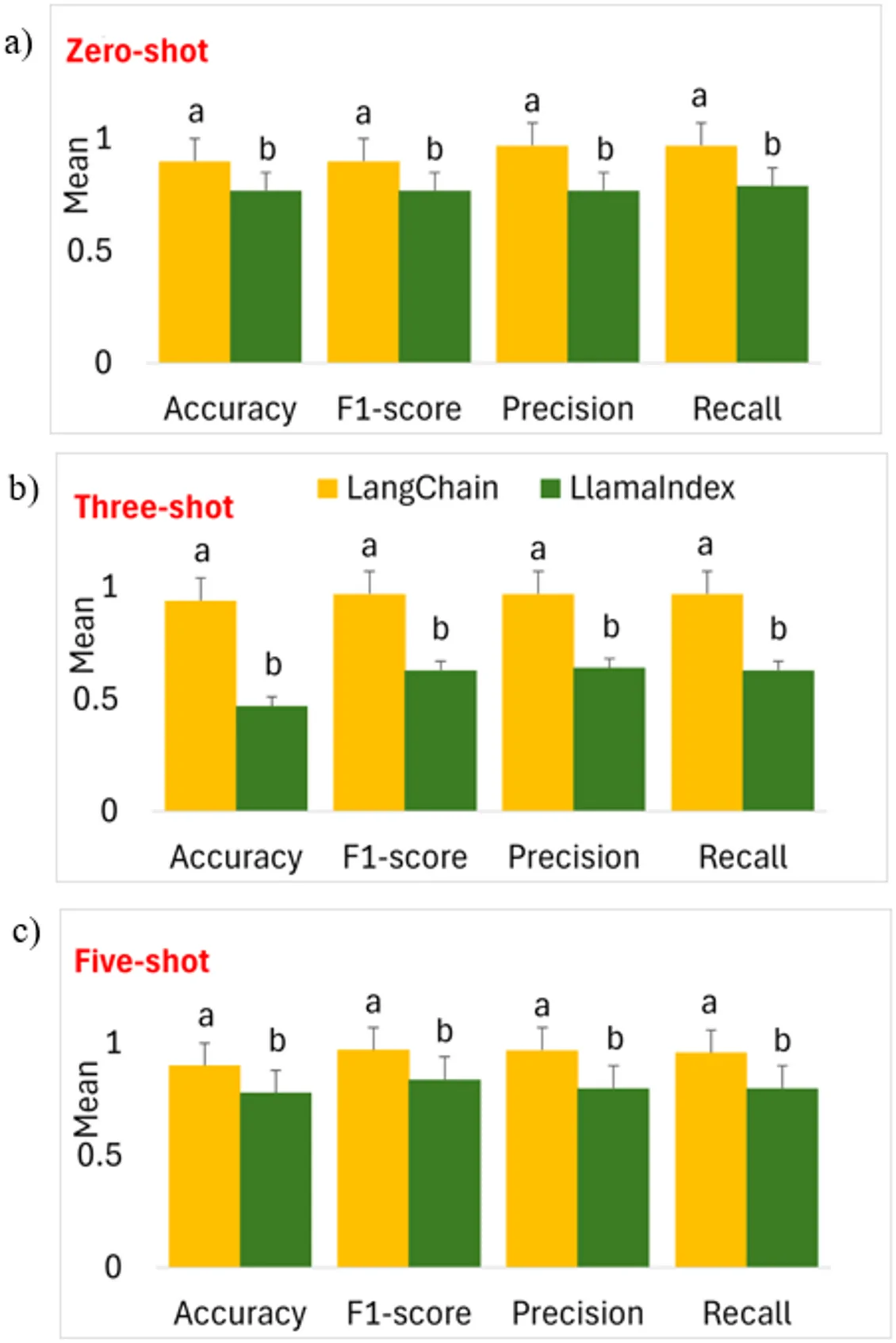
Performance comparison between LangChain and LlamaIndex for the clinical IE using (**a**) zero-shot learning, (**b**) three-shot learning, and (**c**) five-shot learning

In testing Hypothesis 2, we maintained zero-shot, three-shot, and five-shot learning configurations, following the recommendation of Vithanage et al. [5]. The results suggested that the LangChain library significantly outperforms LlamaIndex across three clinical IE tasks in zero-shot, three-shot, and five-shot learning. (see Fig. 2). Specifically, for three-shot learning, moving from LangChain to LlamaIndex caused model accuracy to degrade by 47%, F1-score by 34%, precision by 33%, and recall by 33%. For five-shot learning, LlamaIndex showed a decrease of 12% in accuracy, 13% in F1-score, 17% in precision, and17% in recall. For zero-shot, accuracy decreased by 12%, F1-score by 13%, precision by 20% and recall by 18% compared to LangChain.

These performance differences suggest that LangChain may provide more contextually appropriate retrieval for the clinical IE tasks examined.

#### Discussion

The findings strongly support Hypothesis 2, demonstrating that identical conceptual LLM-RAG architectures can yield substantially different performance outcomes when implemented with LangChain versus LlamaIndex frameworks. The magnitude of performance degradation with LlamaIndex, up to 47% accuracy reduction in 3-shot learning, is practically significant and demands a mechanistic explanation.

We attribute this performance gap to three key architectural differences: (1) the different text chunking strategies. LangChain’s RecursiveCharacterTextSplitter, with custom clinical separators (newlines, periods, and sentence boundaries), preserves semantic coherence by splitting at natural linguistic boundaries. Clinical notes contain complex, multi-sentence observations in which meaning depends on contextual continuity; fragmentation at arbitrary character positions disrupts this continuity. LlamaIndex’s default SentenceSplitter, while effective for well-structured documents, may produce chunks that fragment clinical narratives in ways that obscure relevant information. (2) the retrieval chain architecture. LangChain’s sequential retrieval chain maintains stronger query-document relevance through explicit scoring at each retrieval stage. LlamaIndex’s architecture, optimized for fast document indexing and querying at scale, may sacrifice retrieval precision for throughput, a trade-off that becomes costly in high-precision clinical applications. (3) prompt template handling. LangChain provides more granular control over prompt construction, enabling precise formatting of few-shot examples in ways that may better guide the LLM’s extraction behavior. The substantial performance improvement from zero-shot to three-shot learning observed with LangChain (but not equivalently with LlamaIndex) suggests that LangChain’s prompt handling more effectively leverages in-context learning.

### Testing Hypothesis 3: Increasing the Number of Few-Shot Examples Beyond Three in Prompts Improves the Performance of Clinical Information Extraction in an RAG Architecture

#### Results

**Fig. 3:**
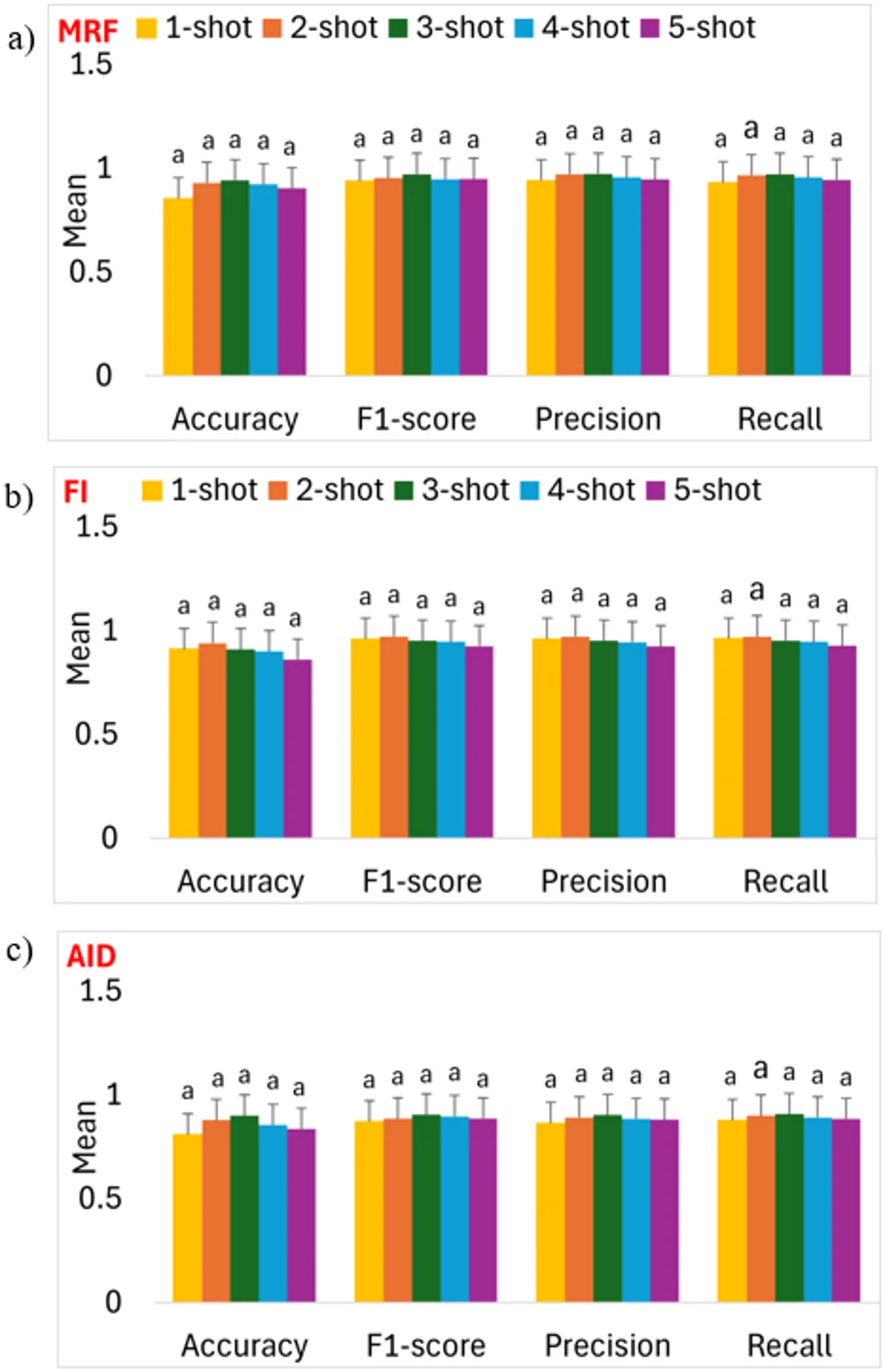
Performance comparison between different few-shot learning examples within the RAG framework for extracting (**a**) frailty index (FI) items, (**b**) malnutrition risk factors (MRF), and (**c**) agitation in dementia (AID)

No statistically significant differences were observed across the one-shot, two-shot, three-shot, four-shot, and five-shot configurations in terms of accuracy, precision, recall, or F1 score for extracting the frailty index, malnutrition risk factors, and agitation in dementia (see Fig. 3). However, a consistent non-significant trend existed that the three-shot prompting configuration outperformed all other settings, including one-shot, two-shot, four-shot, and five-shot configurations, across all tasks. Specifically, it yielded peak results for frailty index extraction (accuracy: 94.00% ± 0.03%, F1: 97.08% ± 0.02%), malnutrition risk factor identification (accuracy: 94.21% ± 0.02%, F1: 97.12% ± 0.02%), and agitation in dementia detection (accuracy: 94.21% ± 0.06%, F1: 97.12% ± 0.03%). These results indicated that while no configuration was significantly superior, 3-shot prompting offered marginal benefits depending on the task.

#### Discussion

The results do not support Hypothesis 3, as increasing the number of few-shot examples beyond three did not further improve clinical IE performance. In contrast, 3-shot prompting marginally outperformed other configurations across all tasks. This finding has important mechanistic and practical implications.

The mechanistic explanation is a fundamental trade-off in in-context learning. With fewer than three examples (1-shot, 2-shot), the model encounters insufficient pattern diversity to generalize across the heterogeneous surface forms of clinical entities. Clinical notes express the same underlying concepts using diverse terminology, abbreviations, and phrasing; a single example cannot capture this variation. Conversely, with more than three examples (4-shot, 5-shot), several factors may limit additional gains: (1) the model approaches its effective context utilization capacity for in-context learning, (2) additional examples may introduce conflicting patterns that confuse rather than guide extraction, and (3) the marginal information gain from each additional example decreases as pattern coverage becomes saturated.

The second possible explanation is the role of the RAG knowledge base. The observed performance plateau beyond three examples may also reflect the interaction between few-shot prompting and RAG retrieval. The RAG knowledge base already provides comprehensive coverage of relevant clinical entity patterns through retrieved passages. In this context, the few-shot examples may primarily serve as a demonstration of the extraction format and task specification rather than to provide exhaustive pattern coverage. Consequently, a small number of well-chosen examples are sufficient to guide the model, while additional examples provide redundant information already available in the retrieved context. The specific optimal number may vary with task complexity, model capacity, and domain characteristics, suggesting that practitioners should empirically validate the optimal example count for their specific applications [34].

Our finding that three examples achieve optimal performance has practical significance for clinical NLP deployment. Constructing high-quality few-shot examples requires clinical expertise and careful annotation, resources that are scarce in healthcare settings. This finding substantially reduces the annotation burden required for deploying RAG-based clinical IE systems. This low-resource learning capability is particularly valuable for extending the system to new clinical domains or institutions where large annotated datasets are unavailable.

### Testing Hypothesis 4: Varying the Number of Top-k Retrieved Documents Impacts the Performance of Clinical Information Extraction in an RAG Architecture

#### Results

**Fig. 4:**
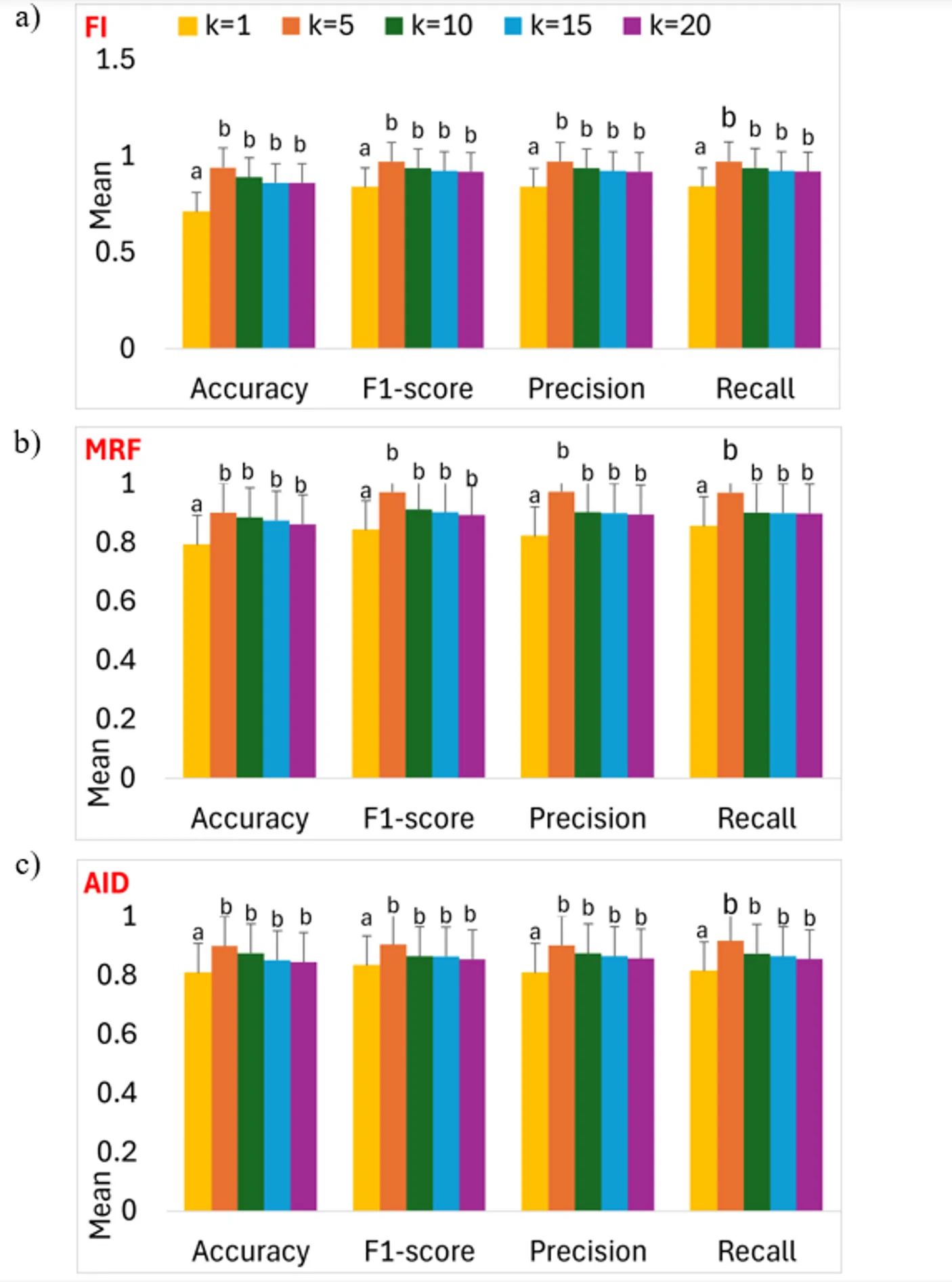
Performance comparison between different numbers of top k within the RAG framework for extracting (**a**) frailty index (FI) items, (**b**) malnutrition risk factors (MRF), and (**c**) agitation in dementia (AID)

To test Hypothesis 4, we systematically varied the number of top-k retrieved documents while maintaining the three-shot learning configuration established in Sect. 3.3. The findings showed that performance improved significantly as the number of top-k retrieved documents increased from 1 to 5 (see Fig. 4). For frailty index extraction, model accuracy improved by 23%, F1-score by 13%, and precision and recall each increased by 13%. In malnutrition risk factor identification, accuracy increased by 11% and F1-score by 13%, along with 15% increases in precision and 11% increases in recall. Similarly, for detecting agitation in dementia, accuracy rose by 10% and F1-score by 10%, with 10% increases in precision and recall, respectively. However, further increases beyond k = 5 (e.g., k = 10, 15, or 20) did not yield statistically significant gains, suggesting that a retrieval depth of 5 was optimal for these tasks.

#### Discussion

The results support Hypothesis 4: varying the number of top-k retrieved documents significantly affects the performance of clinical IE in a RAG architecture. Among the tested values (k = 1, 3, 5, 10, 15, 20), k = 5 yielded the optimal performance, providing sufficient contextual information without overwhelming the model with irrelevant content. The statistically significant improvements from k = 1 to k = 5, up to a 23% increase in accuracy for frailty extraction, represent the largest effect size observed across all experimental conditions, highlighting the critical importance of retrieval depth configuration.

The mechanistic explanation for the k = 5 optimum lies in a fundamental trade-off between retrieval coverage and noise introduction. At k = 1, the system depends entirely on the precision of a single retrieved passage, meaning any retrieval error propagates directly to extraction failure, as reflected in the substantially lower performance at k = 1 across all tasks. At k = 5, the retrieval set provides sufficient redundancy to tolerate individual retrieval errors while maintaining high average relevance, capturing multiple surface realisations of clinical concepts without overwhelming the LLM with irrelevant content.

Beyond k = 5, performance plateaus with no statistically significant gains at k = 10, 15, or 20. This is consistent with the “lost in the middle” phenomenon documented by Liu et al. [35]. It demonstrates that LLM performance degrades when relevant information is positioned in the middle of a long retrieved context rather than at the beginning or end. As additional passages are appended to the context, clinically relevant content retrieved at lower ranks becomes increasingly difficult for the model to attend to effectively, regardless of its semantic relevance. Therefore, including less relevant passages beyond the top 5 introduces noise that dilutes the contextual signal available to the LLM. Moreover, longer context windows may cause the model to disproportionately weigh earlier retrieved passages regardless of their relevance, a pattern consistent with the positional attention bias reported in prior work [35, 36].

In terms of knowledge base characteristics, the optimal k value is likely influenced by knowledge base density and construction. Our relatively compact, curated knowledge bases, where each entry represents a distinct clinical concept mapping, may require fewer retrieved passages than larger, noisier document collections. For knowledge bases with higher redundancy or less precise entries, larger k values might be beneficial, and healthcare organisations should empirically tune this parameter based on their specific knowledge base characteristics.

The k = 5 configuration also offers favourable computational trade-offs. Since retrieval cost scales linearly with k and LLM inference cost scales with total input context length, limiting retrieval to the top 5 passages achieves optimal extraction performance while minimising both retrieval latency and inference costs, an important consideration for production deployment in high-throughput clinical environments.

It is important to acknowledge, however, that the current experiments varied retrieval depth while holding document ordering fixed. As Zhang et al. [36] demonstrated in a medical RAG setting, the position of the most relevant document within the retrieved context can independently affect LLM output quality, even when that document falls within the top-k window. Future work should investigate whether reordering retrieved documents, for instance, placing the most semantically relevant passage at the beginning of the context, yields further performance gains in aged care nursing note IE. Such retrieval-order optimisation represents a complementary and as yet unexplored dimension to top-k tuning in this setting.

### General Discussion

Despite the well-recognized information value of free-text clinical notes in EHRs, extracting information from these notes using LLMs remains challenging [6]. While standard RAG configurations are often optimized for general-purpose information retrieval, they may not transfer to the distinct semantic and structural properties of clinical narratives. This study provides a scientific characterisation of this domain gap, systematically isolating how specific architectural components, from embedding models to retrieval methods and number of prompt learning, impact the accuracy and reliability of clinical information extraction. The scientific value includes: (1) establishing evidence-based configuration guidelines for clinical RAG systems, (2) identifying the structural determinants of performance for varied architectural configurations in clinical contexts, and (3) demonstrating reproducible methodology for clinical NLP benchmarking that can extend to other studies and practical applications.

To date, the clinical NLP literature lacks rigorous comparative studies that isolate the effects of individual RAG components while controlling for confounding variables. Most existing studies either report single configurations without systematic comparison or vary multiple parameters simultaneously, making it impossible to attribute performance differences to specific architectural choices. Our controlled experimental design, which varies one component at a time while holding others constant, enables causal inference about each component’s contribution to overall system performance.

#### Causal Explanations for Component Performance

To define effective generative AI-powered clinical systems, it is important to understand why certain configurations work. This study unveils that the embedding model, BAAI/bge-m3 model, had superior performance compared to other models. This is likely due to its multi-granularity representation learning across diverse multilingual corpora, which may have yielded more robust representations for the heterogeneous writing styles found in clinical notes from residential aged care facilities, which often mix formal health terminology with colloquial expressions. Conversely, models trained exclusively on biomedical literature and healthcare data, such as BioBERT and ClinicalBERT, may fall short at explaining texts that do not fully conform to more scientific, scholarly biomedical standards.

LangChain’s RecursiveCharacterTextSplitter with customized separators enhances semantic preservation by prioritizing splits at natural linguistic boundaries, such as paragraphs and sentences, before applying finer-grained character-level segmentation. This hierarchical strategy is particularly important for clinical notes, where key meaning is often encoded within complete sentences describing symptoms, assessments, or care activities. In contrast, LlamaIndex’s default SentenceSplitter, although effective for well-structured texts, can fragment clinical narratives, disrupting contextual coherence. Additionally, LangChain’s retrieval pipeline typically yields stronger query-document relevance due to its sequential processing architecture, whereas LlamaIndex’s design emphasis on efficient large-scale indexing may introduce trade-offs that reduce retrieval precision.

The effectiveness of 3-shot prompting reflects a fundamental trade-off in in-context learning. With fewer than three examples, the model lacks sufficient pattern diversity to generalize across the heterogeneous expressions of clinical entities. With more than three examples, the prompt approaches the model’s effective context window limit; additional examples yield diminishing returns and may potentially introduce conflicting patterns that confuse the model. Therefore, three examples appear to represent the “Goldilocks zone” where pattern coverage is adequate without exceeding the model’s capacity for in-context abstraction.

The choice of top-k = 5 provides an effective balance between capturing sufficient relevant signal and limiting noise. With k = 1, performance is highly sensitive to retrieval precision, as any single mis retrieval passage directly leads to extraction failure. Increasing to k = 5 introduces useful redundancy: individual retrieval errors are buffered by additional, highly relevant passages, preserving overall contextual integrity. Larger values of k admit lower-relevance or off-topic passages, which can dilute the contextual signal and introduce contradictions that impair extraction accuracy. The optimal k value likely depends on the density and quality of the knowledge base; in our case, the compact and curated nature of the clinical knowledge sources reduces the need for larger retrieval sets.

#### Interaction Effects Between Components

An important finding is that component effects are not fully independent. The LangChain + bge-m3 combination exhibited synergistic performance exceeding the sum of their individual contributions. This synergy may arise because bge-m3’s multi-granularity representations align well with LangChain’s hierarchical text processing, creating complementary semantic representations at the embedding and chunking levels. Future work should explicitly model these interaction effects through factorial experimental designs.

#### Implications for Clinical Information Retrieval

This research’s findings have the following implications for clinical information retrieval. First, they provide evidence-based configuration recommendations that can be deployed immediately with open-source tooling, reducing the trial-and-error burden typical in clinical NLP pipelines. The optimal configuration (LangChain + BAAI/bge-m3 + 3-shot + top-k = 5) achieved F1 scores exceeding 90% across all three clinical IE tasks, meeting performance requirements for clinical decision support applications where high precision is essential to minimizing false-positive alerts.

Second, the strong performance of the three-shot prompting strategy demonstrates that high-quality extraction can be achieved without large annotated datasets or model fine-tuning. This is particularly advantageous in clinical settings where labeled training data are scarce due to privacy restrictions, annotation costs, and the specialized expertise required for reliable clinical labeling.

Third, accurate entity extraction supports automated structuring of free-text clinical notes, reducing documentation burden and facilitating earlier identification of clinical deterioration. In aged care contexts, reliable detection of frailty indicators, malnutrition risk factors, and agitation symptoms can enable more proactive clinical intervention.

Fourth, the computational efficiency of the optimal configuration, combining moderate top-k retrieval with few-shot prompting rather than fine-tuning, supports scalability for large-volume document processing, a key requirement for real-world clinical deployment in resource-constrained healthcare and aged care settings.

Finally, while our study focused on aged care documentation, the multilingual and multi-granularity representations of the BAAI/bge-m3 model suggest broader applicability across institutions with documentation styles. Nevertheless, external validation across different EHR systems remains necessary to confirm generalizability.

### Study Limitations and Future Work

This study has several limitations that should be acknowledged. The clinical IE evaluation was focused on three specific tasks from residential aged care settings: extracting frailty index items, identifying malnutrition risk factors, and detecting agitation in dementia. While these tasks are clinically meaningful and represent diverse extraction challenges (multi-label classification, risk factor identification, symptom detection), they may not fully capture the variability of clinical documentation across acute care, outpatient, or speciality settings. Future work should validate these findings across broader healthcare environments, including emergency departments, intensive care units, and primary care, where documentation styles and clinical terminology differ substantially.

While we systematically varied embedding models, retrieval frameworks, top-k values, and few-shot counts, several potentially important configuration parameters were held constant or not fully explored. We did not change other default settings. These include chunk size and overlap parameters within each framework; similarity score thresholds for retrieval filtering; alternative vector database implementations beyond FAISS; temperature and other LLM generation parameters; or prompt template structures beyond few-shot count variation. Each of these parameters may interact with the variables we manipulated, and future factorial designs should systematically explore these interactions.

Our experiments used only Llama-3.1-8B-Instruct due to practical constraints (GPU availability, regulatory compliance, reproducibility). However, different LLMs may exhibit varying sensitivity to RAG configurations. Larger models may tolerate higher k values without performance degradation; instruction-tuned models may require fewer few-shot examples; and models with longer context windows may benefit from different chunking strategies. Future work should replicate these experiments across multiple LLMs (e.g., GPT-4, Claude, Mistral, Gemma) to establish the generalizability of optimal configurations.

This study evaluated only in-context learning approaches (few-shot prompting with RAG) and did not compare them with fine-tuned models. While our goal was to identify optimal configurations for deployment scenarios where fine-tuning is impractical due to limited labelled data, computational resource constraint, and regulatory constraints, understanding the performance ceiling achievable with fine-tuning would contextualise the practical significance of our findings.

Several comparisons, particularly between embedding models, did not reach statistical significance despite consistent rank ordering across tasks. This may reflect insufficient sample sizes for detecting moderate effect sizes. Future studies should conduct power analyses to ensure adequate sample sizes for detecting clinically meaningful performance differences.

While we observed synergistic effects between certain component combinations (e.g., LangChain + bge-m3), our one-at-a-time experimental design cannot fully characterize higher-order interaction effects. Future work employing full- or fractional-factorial designs would enable a more comprehensive understanding of component interactions.

The retrieval module in this study relies solely on dense embedding-based representations, which, while effective for capturing semantic similarity, may not fully preserve fine-grained lexical nuances present in aged care nursing notes. Sparse retrieval methods such as BM25 offer complementary advantages, particularly for matching exact abbreviations and domain-specific shorthand that may be underrepresented in embedding spaces. Hybrid retrieval approaches that combine sparse and dense methods are increasingly common in real-world RAG deployments and represent a promising direction for improving retrieval precision in clinical IE tasks. We identify the exploration of hybrid retrieval configurations as an important avenue for future work.

This study evaluated a single model (Llama-3.1-8B-Instruct), which may limit the generalisability of the findings. We acknowledge this as a constraint of the current work. Nevertheless, investigating optimal hyperparameters for small, instruction-tuned models is itself a valuable contribution, as many academic and clinical research settings lack the GPU resources required to run larger models. Future studies will extend this framework to additional small open-source models (e.g., Phi-3, Mistral-7B) and, where resources permit, proprietary models such as GPT-4 and Claude, to assess the transferability of the optimal parameter configurations identified here.

All experiments used clinical notes from a single Australian aged care organisation. Documentation practices, clinical terminology, and EHR systems vary substantially across healthcare organizations and countries. The generalizability of optimal configurations to other settings requires external validation studies.

## Conclusion

This study systematically explored the impact of varying individual components within the RAG architecture to identify the most effective configuration for clinical information extraction (IE) from an LLM. The evaluation focused on extracting key indicators, such as the frailty index, malnutrition risk factors, and agitation in dementia, from clinical notes. Through a series of rigorously designed experiments testing four research hypotheses, we evaluated how variations in embedding models, RAG configurations, retrieved top-k parameters, and few-shot prompt numbers affect clinical information extraction performance. The results highlight the importance of systematically optimizing RAG components to improve LLM-integrated clinical IE systems. These findings offer practical, evidence-based guidance for designing effective RAG-based architectures in LLM-based clinical information retrieval applications. These insights offer meaningful guidance for future research and deployment of LLM-powered clinical IE solutions, particularly in handling free-text data within sensitive and high-stakes healthcare environments.

## Electronic Supplementary Material

Below is the link to the electronic supplementary material.

Supplimentray File Q5

## Data Availability

Data is not available to comply with the Australian laws on privacy protection.

## Declarations

This manuscript has not been published elsewhere and is not under consideration by another journal. All authors have approved the manuscript and agree with its submission to the Journal.
