# Supplementary material for: Evaluating RAG Configurations for Clinical Information Extraction from EHR Notes: Aged Care Case Study
Source: J Healthc Inform Res. 2026 Sep 14;10(4):952–72. doi: 10.1007/s41666-026-00254-8 (PMC13719912; doi:10.1007/s41666-026-00254-8)
Supplement: Supplementary file 1 [file 41666_2026_254_MOESM1_ESM.docx]

**Evaluating RAG Configurations for Clinical Information Extraction from EHR Notes: Aged Care Case Study**

Dinithi Vithanage^1^, Quang Vinh Duong^1^, Chao Deng^2^, Rajendra Acharya^3^, Zhanzhong Gu, Ting Song^1^, Ping Yu^1, *^

1 School of Computing and Information Technology, University of Wollongong, Wollongong, Australia
2 School of Medical, Indigenous and Health Sciences, University of Wollongong, Wollongong, Australia
3 School of Mathematics, Physics and Computing, University of Southern Queensland

***Correspondence:** ping@uow.edu.au

**Supplementary Table 1. Clinical tasks with their labels and frequency**.

| Clinical Tasks | Labels | Number of Clinical Entities |
| --- | --- | --- |
| Frailty Index | Activity limitation, anaemia and haematinic deficiency, arthritis, atrial fibrillation, cerebrovascular disease, chronic kidney disease, diabetes, dizziness, dyspnoea, falls, foot problems, fragility fracture, hearing impairment, heart failure, heart valve disease, housebound, hypertension, hypotension/syncope, ischaemic heart disease, memory and cognitive impairment, mobility and transfer problems, osteoporosis, Parkinsonism and tremor, peptic ulcer, peripheral vascular disease, polypharmacy, requirement for care, respiratory disease, skin ulcer, sleep disturbance, social vulnerability, thyroid disease, urinary incontinence, urinary system disease, visual impairment, weight loss and anorexia | 36 |
| Malnutrition risk factors | Pain, panamax, hydrozole, skin blanching, excoriation, mobility and care dependency, reduced mobility, sustagen, frailty, dementia, lymphoma, fracture, macular degeneration, unsteady gait, fall risk, temaze, anxiety, worry, movicol, temazepam, pruritus, agitation, distress, paracetamol, analgesics, insomnia, eating dependency, fluid intake deficiency, cognitive disorder, fall, poor vision, hearing loss, bruise, wound, homesickness, refusing medications, dyspnea, lack of insight, blood glucose, chronic obstructive pulmonary disease, gastroesophageal reflux disease, hypertension, osteoarthritis, stroke, food modification, fluid modification, dry skin, pressure ulcer, alcohol use disorder, dietary restriction, history of malnutrition, nutritional supplement, vitamin b12, skin redness, confusion, acute kidney injury, age related decline, urinary tract infection, myelodysplastic syndrome, type 2 diabetes, hypothyroidism, depression, seizure disorder, weight loss, decreased appetite, double incontinence, hypoglycemia, deteriorating condition, mood change, discomfort, osteo paracetamol, injury, febridol, broken skin, nail abnormality, prostate cancer, psychosis, sadness, kyphosis, social isolation, oral health issue, chest pain, back pain, suboptimal intake, nutritional risk assessment, poor balance, reduced range of motion (rom), shortness of breath (sob), paranoia, urinary incontinence, frailty, fatigue, irritable bowel syndrome, neuro bas, indwelling catheter, restlessness, decreased appetite, disorientation to person, disorientation to time, disorientation to place, ordine, risperidone, delusion, laceration, lethargy, low spo2, malaise, clammy skin, vasovagal event, cough, fever, antibiotics, weakness, chest infection, palliative care status, underweight, resolve plus cream, hydrocortisone cream, skin infection, allergic rhinitis, aricept, augmentin, body weight, bowel cancer, buscopan, emycin, endone, enprocal, gaviscon, iv fluids, invasive ductal carcinoma, madopar, nsaids, nephrectomy, nero bas, nexium, norspan patch, ordine, palliative care, sifrol, spiriva handihaler, stalevo, tragin, ventolin, xarelto, insulin, anticoagulant, antihistamines, antroquoril cream, aperients, arginaid, aspirin, bactroban cream, benzodiazepines, candacort cream, claratyne, cubitan, diaformin, diuretic medication, doxycycline, duloxetine, efudix, emollient, esomeprazole, gabapentin, linagliptin, maxolon, metformin, mirtazapine, morphine, opiates, osmolax, oxycontin, pain patch, panadol, prednisolone, statin, olanzapine, amoxil, quetiapine, targin, syquet, oral health issue, social isolation, abdominal obesity, activities of daily living score, anaemia, anorexia, anticholinergic burden, apathy, argumentative behaviour, arrhythmia, ascites, aspiration, aspiration pneumonia, asthma, at risk of malnutrition, b12 deficiency, bipolar disorder, bladder obstruction, blepharitis, blindness, bowel incontinence, bowel irregularity, bowel obstruction, bowel resection, burn, cachexia, cancer, candidiasis, cardiomyopathy, celiac disease, change in dietary behaviour, choking, chronic ear perforation, chronic kidney disease, chronic pancreatitis, cognitive assessment score, colorectal disorder, colostomy, complaint with meal, contracture, deafness, deficit in motor planning and initiation, dehydration, delirium, delirium episode, depression, desquamation, diabetes, diarrhea, digestive issue, distended abdomen, diverticular disease, diverticulitis, downing effect, drowsiness, dysuria, eating disorder, eating too quickly, eczema, endstagerenaldisease, environmental hazard, epilepsy, faecal smearing, fat loss, fecal vaginal contamination, feel down, food hoarding, food intolerance, food preference, forgetting to eat, gait disturbance, gastritis, gastroparesis, gastrostomy, giddiness, gout, grief, hiv, haematemesis, hallucination, heart disease, heart failure, hiatal hernia, high output ileostomy, hopelessness, hyperactivity, hyperlipidemia, hyperthyroidism, hypomagnesemia biochemical, hypotension, ileostomy, inflammatory bowel disease, intentional weight loss, intestinal stricture, iron deficiency, irregular bowels, ischemic disease, lactose intolerance, liver disease, loss of smell, losse bowel, low bmi, low level of consciousness, lung nodule, malabsorption, meal time anxiety, medication non-adherence, memory impairment, mentally dull, mixed anxiety and depressive disorder, multiple sclerosis, muscle wasting, muscular dystrophy, nausea, nephrolithiasis, neurological disorder, not responding to any command, not tolerating current diet, not verbal, oedema, osteomalacia, osteoporosis, overactive bladder syndrome, pale pallor, paralysis, parkinson disease, peptic ulcer, peripheral vascular disease, pleural effusion, pneumonia, polypharmacy, pooling food in mouth, poor coordination, poor voluntary muscle control, post traumatic stress disorder, prolonged aptt, pulmonary fibrosis, rectal prolapse, self harming behaviour, sepsis, shallow breathing, short bowel syndrome, sick feeling, smoking, spitting out food, stroke residual effects, suicidality, sundowner syndrome, suspicious about food, temporomandibular disorder, thyroid disorder, tinnitus, tremor, umbilical hernia, unfit denture, unintentional weight loss, upper respiratory tract infection, urosepsis, valvular heart disease, venous ulcer, vertigo, vitamin D deficiency, volvulus, vomiting, wandering | 320 |
| Agitation in dementia | Disruptive vocalisation, verbally aggressive behaviour, arguing, complaining, cursing, threat, using abusive language, using accusatory language, using foul language, using hostile language, using obscene language, using profane language, verbally nonaggressive behaviour, ceaseless talking, constant repetition of word, constant unwarranted requests for attention, constant unwarranted requests for help, constant unwarranted requests for reassurance, echolalia, groaning, grunting, howling, making bizarre noise, rambling, repetitive questioning, roaring, screaming, shouting, speaking in excessively loud voice, emotional distress, anger, frustration, irritability, mood swing, negativism, outburst, physically aggressive behaviour, biting, destroying property, fighting, grabbing, hitting, hurting self, hurting someone, kicking, pushing, resisting, scratching, shoving, slamming, spitting on people, staring, striking people, tearing, throwing object, physically nonaggressive behaviour, constant manipulation of object, fidgeting, frightening others, gesturing, hand wringing, inappropriate dressing, inappropriate handling object, inappropriate undressing, pacing, pointing finger, repetitive physical mannerism, restlessness, rocking, rummaging, searching, wandering, bruxism, resisting, punching, absconding, calling out, physical agitation, facial grimacing, paranoia, moving objects, hoard items, intrusive of others privacy, gets up and down from constantly, urinating on the floor. | 85 |

**Supplementary Table 2. Prompts used in the study.**

| Frailty Index | <\|begin_of_text\|><\|start_header_id\|>system<\|end_header_id\|>  You are an assistant that extracts frailty index items from a given electronic health record.  Your answer must be based solely on the following context: {context}  <\|eot_id\|>  <\|start_header_id\|>user<\|end_header_id\|>  Extract frailty index items from this electronic health record: {question}  You only extract outputs based on 35 frailty index items given in this list: ['activity limitation', 'anaemia and haematinic deficiency', 'arthritis', 'atrial fibrillation', 'cerebrovascular disease', 'chronic kidney disease', 'diabetes', 'dizziness', 'dyspnoea', 'falls', 'foot problems', 'fragility fracture', 'hearing impairment', 'heart failure', 'heart valve disease', 'housebound', 'hypertension', 'hypotension or syncope', 'ischaemic heart disease', 'memory and cognitive problems', 'mobility and transfer problems', 'osteoporosis', 'parkinsonism and tremor', 'peptic ulcer', 'peripheral vascular disease', 'polypharmacy', 'respiratory disease', 'skin ulcer', 'sleep disturbance', 'social vulnerability', 'thyroid disease', 'urinary incontinence', 'urinary system disease', 'visual impairment', 'weight loss and anorexia']  Please do not explain for the answer, just generate output only and the output must come from 35 given frailty indexes list.  If you don't know the answer, return "no symptom". If the answer is "no symptom", NO other text MUST be included.  Extract only direct frailty index items explicitly stated in the electronic health record. Do not include inferred or implied entities.  The answer format must be in format like this: item1, item2, item3,...  Example 1: appears to have suffered no ill effects from fall yesterday, staff fed his meals. Observations attended and stable. has not displayed any signs of pain.  Answer 1: no symptom  Example 2: The resident's BGL checked before dinner. 18.8mmol. On the range. Sliding scale 8 units insulin given as charted. Medication given as charted. Had his dinner, staffs to encourage the resident to drink water instead of orange juice. Nil complain of feeling dizziness, nausea or vomiting episode this shift. Alert and orientated to time, place and person. Normal breathing and normal color of the face. Pressure area care attended. Pad changed and perianal care attended. IDC insitu; patent and draining well. Settled and comfortable ATOR.  Answer 2: diabetes, urinary incontinence  <\|eot_id\|>  <\|start_header_id\|>assistant<\|end_header_id\|>  """ |
| --- | --- |
| Malnutrition Risk Factors | <\|begin_of_text\|><\|start_header_id\|>system<\|end_header_id\|>  An electronic health record is given from user.  Given list of malnutrition risk factors:["Pain", "Panamax", "hydrozole", "Skin Blanching", "Excoriation", "Mobility And Care Dependency", "Reduced Mobility", "Sustagen", "Frailty", "Dementia", "Lymphoma", "Fracture", "Macular Degeneration", "Unsteady Gait", "Fall Risk", "Temaze", "Anxiety", "Worry", "Movicol", "Temazepam", "Pruritus", "Agitation", "Distress", "paracetamol", "analgesics", "Insomnia", "Eating Dependency", "Fluid Intake Deficiency", "Cognitive Disorder", "Fall", "Poor Vision", "Hearing Loss", "Bruise", "Wound", "Homesickness", "Refusing Medications", "Dyspnea", "Lack Of Insight", "Blood Glucose", "Chronic Obstructive Pulmonary Disease", "Gastroesophageal Reflux Disease", "Hypertension", "Osteoarthritis", "Stroke", "Food Modification", "Fluid Modification", "Dry Skin", "Pressure Ulcer", "Alcohol Use Disorder", "Dietary Restriction", "History Of Malnutrition", "Nutritional supplement", "Vitamin B12", "Skin Redness", "Confusion", "Acute Kidney Injury", "Age Related Decline", "Urinary Tract Infection", "Myelodysplastic Syndrome", "Type 2 Diabetes", "Hypothyroidism", "Depression", "Seizure Disorder", "Weight Loss", "Decreased Appetite", "Double Incontinence", "Hypoglycemia", "Deteriorating Condition", "Mood Change", "Discomfort", "Osteo paracetamol", "Injury", "Febridol", "Broken Skin", "Nail Abnormality", "Prostate Cancer", "Psychosis", "Sadness", "Kyphosis", "Social Isolation", "Oral Health Issue", "Chest Pain", "Back Pain", "Suboptimal Intake", "Nutritional Risk Assessment", "Poor Balance", "Reduced Range of Motion (ROM)", "Shortness of Breath (SOB)", "Paranoia", "Urinary Incontinence", "Frailty", "Fatigue", "Irritable Bowel Syndrome", "Neuro Bas", "Indwelling Catheter", "Restlessness", "Decreased Appetite", "Disorientation To Person", "Disorientation To Time", "Disorientation To Place", "Ordine", "Risperidone", "Delusion", "Laceration", "Lethargy", "Low SpO2", "Malaise", "Clammy Skin", "Vasovagal Event", "Cough", "Fever", "Antibiotics", "Weakness", "Chest Infection", "Palliative Care Status", "Underweight", "Resolve Plus cream", "Hydrocortisone Cream", "Skin Infection", "Allergic Rhinitis", "Aricept", "Augmentin", "Body Weight", "Bowel Cancer", "Buscopan", "Emycin", "Endone", "Enprocal", "Gaviscon", "IV fluids", "Invasive Ductal Carcinoma", "Madopar", "NSAIDs", "Nephrectomy", "Nero bas", "Nexium", "Norspan patch", "Ordine", "Palliative Care", "Sifrol", "Spiriva handihaler", "Stalevo", "Tragin", "Ventolin", "Xarelto", "insulin", "anticoagulant", "antihistamines", "antroquoril cream", "aperients", "arginaid", "aspirin", "bactroban cream", "benzodiazepines", "candacort cream", "claratyne", "cubitan", "diaformin", "diuretic medication", "doxycycline", "duloxetine", "efudix", "emollient", "esomeprazole", "gabapentin", "linagliptin", "maxolon", "metformin", "mirtazapine", "morphine", "opiates", "osmolax", "oxycontin", "pain patch", "panadol", "prednisolone", "statin", "Olanzapine", "Amoxil", "Quetiapine", "Targin", "Syquet", "Oral Health Issue", "Social Isolation", "Abdominal Obesity", "Activities Of Daily Living Score", "Anaemia", "Anorexia", "Anticholinergic Burden", "Apathy", "Argumentative Behaviour", "Arrhythmia", "Ascites", "Aspiration", "Aspiration Pneumonia", "Asthma", "At Risk Of Malnutrition", "B12 Deficiency", "Bipolar Disorder", "Bladder Obstruction", "Blepharitis", "Blindness", "Bowel Incontinence", "Bowel Irregularity", "Bowel Obstruction", "Bowel Resection", "Burn", "Cachexia", "Cancer", "Candidiasis", "Cardiomyopathy", "Celiac Disease", "Change In Dietary Behaviour", "Choking", "Chronic Ear Perforation", "Chronic Kidney Disease", "Chronic Pancreatitis", "Cognitive Assessment Score", "Colorectal Disorder", "Colostomy", "Complaint With Meal", "Contracture", "Deafness", "Deficit In Motor Planning And Initiation", "Dehydration", "Delirium", "Delirium Episode", "Depression", "Desquamation", "Diabetes", "Diarrhea", "Digestive Issue", "Distended Abdomen", "Diverticular Disease", "Diverticulitis", "Downing Effect", "Drowsiness", "Dysuria", "Eating Disorder", "Eating Too Quickly", "Eczema", "EndStageRenalDisease", "Environmental Hazard", "Epilepsy", "Faecal Smearing", "Fat Loss", "Fecal Vaginal Contamination", "Feel Down", "Food Hoarding", "Food Intolerance", "Food Preference", "Forgetting To Eat", "Gait Disturbance", "Gastritis", "Gastroparesis", "Gastrostomy", "Giddiness", "Gout", "Grief", "HIV", "Haematemesis", "Hallucination", "Heart Disease", "Heart Failure", "Hiatal Hernia", "High Output Ileostomy", "Hopelessness", "Hyperactivity", "Hyperlipidemia", "Hyperthyroidism", "Hypomagnesemia Biochemical", "Hypotension", "Ileostomy", "Inflammatory Bowel Disease", "Intentional Weight Loss", "Intestinal Stricture", "Iron Deficiency", "Irregular Bowels", "Ischemic Disease", "Lactose Intolerance", "Liver Disease", "Loss Of Smell", "Losse Bowel", "Low BMI", "Low Level Of Consciousness", "Lung Nodule", "Malabsorption", "Meal Time Anxiety", "Medication Non-Adherence", "Memory Impairment", "Mentally Dull", "Mixed Anxiety And Depressive Disorder", "Multiple Sclerosis", "Muscle Wasting", "Muscular Dystrophy", "Nausea", "Nephrolithiasis", "Neurological Disorder", "Not Responding To Any Command", "Not Tolerating Current Diet", "Not Verbal", "Oedema", "Osteomalacia", "Osteoporosis", "Overactive Bladder Syndrome", "Pale Pallor", "Paralysis", "Parkinson Disease", "Peptic Ulcer", "Peripheral Vascular Disease", "Pleural Effusion", "Pneumonia", "Polypharmacy", "Pooling Food In Mouth", "Poor Coordination", "Poor Voluntary Muscle Control", "Post Traumatic Stress Disorder", "Prolonged APTT", "Pulmonary Fibrosis", "Rectal Prolapse", "Self Harming Behaviour", "Sepsis", "Shallow Breathing", "Short Bowel Syndrome", "Sick Feeling", "Smoking", "Spitting Out Food", "Stroke Residual Effects", "Suicidality", "Sundowner Syndrome", "Suspicious About Food", "Temporomandibular Disorder", "Thyroid Disorder", "Tinnitus", "Tremor", "Umbilical Hernia", "Unfit Denture", "Unintentional Weight Loss", "Upper Respiratory Tract Infection", "Urosepsis", "Valvular Heart Disease", "Venous Ulcer", "Vertigo", "Vitamin D Deficiency", "Volvulus", "Vomiting", "Wandering"].  Answer the question based on your knowledge. Use the following context to help:  Example 1: Due to Parkinson disease progression showing aggressive unmanageable behaviours hostile towards staffs kicking legs and hitting on arms started half hour after partner left at 2000 hour,very delhusional hallucinating disorientated to time to place very risky behaviour to himself and to staffs running in corridor intrusive getting into resident rooms and interfering will not sit or stand still offered food and drink refused tried to toilet him same result refusal called partner as soon as heard his voice he calm down and settled a bit then started again looking for door to leave the floor partner came and put him to bed and told him to stay in bed ? behaviour escalating due to sundowner syndrome aggravated by parkinson by infection source will need to reassess condition and find more suitable place or way to manage behaviour risk of head injury, of fracture an accident waiting to happen x2 nurses to attend at all time constantly monitored.  Answer 1: Parkinson Disease, Agitation, Delusion, Hallucination, Disorientation To Time, Disorientation To Time, Restlessness, Refusal To Eat, Refusal Of Care, Sundowner Syndrome  Example 2: Resident faces challenges with poor balance and is at risk of falls due to impulsivity and reduced balance. Staff supervise resident's transfers and mobility using a 4-wheelie walker. As resident's dementia progresses, she tends to wander into other residents' rooms, mistakenly believing they occupy her bed. This behaviour frightens other residents, resulting in arguments. Staff frequently intervene, redirect resident, and provide extra reassurance and diversion throughout the day. As her dementia advances, resident lacks insight into her care needs. She adamantly refuses staff assistance changing her continence aids and attending to her hygiene. Due to this progression, staff guide her through mealtimes, set the table, provide cutlery, and supervise and encourage her during meals and drinks.  Answer 2: wandering, frightening others, refusing care, and arguing  Example 3: is diagnosed with Parkinson and dementia. has history of recurrent falls that usually happens in the evening and night. Since, had 13 falls. have fluctuating mobility. Sometimes he is so stiff and rigid (due to parkinson) that makes it difficult for staff to move him and at times, he is quite flexible that he moves by himself. tends to mobilise on his own hence reason for his falls. A sensor mat was put in place in his room to monitor if is up from his bed. A crash mat is also insitu. also is on visual check, for staff to regularly check on him. toilets regularly to minimise occurrence of incontinence and fall (probably a reason why tends to mobilise). requires 2-3 person assisst for his mobility. is unable to to follow commands due to poor comprehension secondary to dementia. requires assistance with all his ADLs.  Answer 3: Parkinson Disease, Dementia, Fall, Reduced Mobility, Mobility And Care Dependency  Example 4: Resident requires the assistance of a nurse for his hygiene and toileting needs, primarily due to his unsteady gait. He utilises pads due to incontinence issues. Additionally, he is prone to constipation and receives aperients as necessary. The current intervention measures in place have proven to be effective.  Answer 4: constipation.  {context}  <\|eot_id\|>  <\|start_header_id\|>user<\|end_header_id\|>  Please do not explain for the answer, just generate output only and the output must be one of given malnutrition risk factors.  {question}  <\|eot_id\|>  <\|start_header_id\|>assistant<\|end_header_id\|> |
| Agitation in Dementia | <\|begin_of_text\|><\|start_header_id\|>system<\|end_header_id\|>  Nursing notes are given from user.  Given a list of Agitation Symptoms: ["Wandering", "bruxism", "making musical, repeated sound", "physical agitation/aggression", "verbally disruptive", "calling out", "screaming", "delusion", "frightening others", "refusal of care", "arguing", "crying", "restless", "grabbing staff", "spitting out", "groaning", "grimacing", "constant talking", "paranoia", "moving objects", "repeating", "disorientation to time and place", "frequent movements in bed", "resistive", "physically aggressive", "scratching", "disruptive", "picking up items that do not belong to her", "disturbing other residents", "constant noise", "yelling", "refusing meal", "verbally abusive", "shouting", "throwing", "tearful", "interrupt other residents", "using inappropriate words", "physical violence", "pacing", "facial grimacing", "moaning", "irritable", "pinch", "teary", "refuses ADLs", "tapping", "constantly picking", "walks up and down", "removes clothing and pads", "argumentative", "visual hallucination", "rocking", "shout", "smack at staff", "push", "vocal abusive", "throwing object", "declining medication", "urinating on the floor", "negativism", "compliant", "get up at mid night and asking staff to take her to breakfast", "growling", "goes into other resident's rooms", "fiddling", "using hostile language", "mood swing", "destroying property", "unpack her entire wardrobe onto her bed", "communicates in own language in a loud voice", "swearing", "slapping", "pulling", "tripping", "faecal smears", "repetitive questioning", "self harm behaviour", "consistent unwarranted requests for help", "consistent repetition of word", "cranky", "short tempered", "ringing at inappropriate times", "lock the door", "barricade the door with furniture", "making loud noises", "breaking of three windows", "behavioural outbursts", "anxiety", "playing with food if left alone", "difficulty in communicating", "resistance to care", "communication impairment", "ask about her family and home", "abscond", "disorientated", "refuse to be feed by staff", "constantly trying to get up from her chair", "limited communication", "limited comprehension", "anxious", "unable to communicate needs", "poor comprehension", "poor cognition", "push, “hit”", "slapping", "making a mess", "confusion", "non verbal signs of pain", "poor short term memory", "abusive towards staff and other residents", "distracted", "argues", "hide", "constantly repeats", "hums", "inability to concentrate", "rude", "resistant to staff assistance", "spit out medication", "Resist medication", "aggression", "verbally angry", "physically banging on tables", "threatening", "repeatedly asking the same questions", "complaining", "emotional distress", "absconding", "constant unwarranted requests for reassurance", "verbally aggressive behaviour", "cursing", "using abusive language", "using accusatory language", "using foul language", "using obscene language", "using profane language", "verbally nonaggressive behaviour", "ceaseless talking", "constant repetition of word", "constant unwarranted requests for attention", "echolalia", "grunting", "howling", "making bizarre noise", "rambling", "roaring", "speaking in excessively loud voice", "anger", "frustration", "outburst", "biting", "fighting", "hurting self", "hurting someone", "kicking", "shoving", "slamming", "spitting on people", "staring", "striking people", "constant manipulation of object", "fidgeting", "gesturing", "hand wringing", "inappropriate dressing", "inappropriate handling object", "inappropriate undressing", "pointing finger", "repetitive physical mannerism", "rummaging", "searching", "punching", "hoard items", "intrusive of others privacy", "gets up and down from constantly"]  Answer the question based on your knowledge. Use the following context to help:  Example 1: Resident, who suffers from vascular dementia, displays confusion, disorientation, and notable physical agitation/aggression and verbal behaviours. Resident exhibits verbal disruption, calling out and screaming, which disturbs others. Resident frequently requires staff assistance for reassurance, comfort, and distraction using strategies such as music therapy, playing cards, or engaging in simple puzzles. Additionally, when highly distressed, staff contact her daughter to speak with her for comforting purposes. Although she can follow simple instructions, at times, she experiences considerable distress.  Answer 1: Agitation symptoms: physical agitation/aggression and verbal behaviors, including verbal disruption, calling out, and screaming  Example 2: Resident faces challenges with poor balance and is at risk of falls due to impulsivity and reduced balance. Staff supervise resident's transfers and mobility using a 4-wheelie walker. As resident's dementia progresses, she tends to wander into other residents' rooms, mistakenly believing they occupy her bed. This behaviour frightens other residents, resulting in arguments. Staff frequently intervene, redirect resident, and provide extra reassurance and diversion throughout the day. As her dementia advances, resident lacks insight into her care needs. She adamantly refuses staff assistance changing her continence aids and attending to her hygiene. Due to this progression, staff guide her through mealtimes, set the table, provide cutlery, and supervise and encourage her during meals and drinks.  Answer 2: Agitation symptoms: wandering, frightening others, refusing care, and arguing  Example 3: Resident has recently become more physically aggressive due to advancing dementia. During interactions with staff, she sometimes lashes out by punching, especially when feeling threatened or confused. Staff members are providing additional reassurance to minimize these occurrences and maintain a safe environment for all residents.  Answer 3: Agitation symptoms: physically aggressive (lashes out with punching)  Example 4: Resident attended post-hospital discharge and was sent off to pathology, awaiting results. Wandering around. Eating well this shift. Needs privacy and dignity at meal time.  Answer 4: Agitation symptoms: Wandering  Example 5: Request by nursing home for contribution to a resident care plan. Advanced dementia. Bruxism. Resistive to care. Extensive DVT in leg; on long-term NOACs. Requires assistance with foot care.  Answer 5: Agitation symptoms: Bruxism, Resistive to care  {context}  <\|eot_id\|>  <\|start_header_id\|>user<\|end_header_id\|>  Please do not explain for the answer, just generate output only. If none are found, respond with "none".  {question}  <\|eot_id\|>  <\|start_header_id\|>assistant<\|end_header_id\|> |

**Supplementary Table 3.**

**Experimental results are presented as mean values with corresponding standard deviations.**

| Clinical Task | Method | Accuracy | F1-score | Precision | Recall |
| --- | --- | --- | --- | --- | --- |
| Frailty index | Alibaba-NLP/gte-large-en-v1.5 | 88.21% (± 0.013%) | 89.95% (± 0.018%) | 89.96% (± 0.018%) | 89.93% (± 0.019%) |
|  | BAAI/bge-largeen-v1.5 | 90.41% (± 0.035%) | 92.24% (± 0.01%) | 92.24% (± 0.01%) | 92.24% (± 0.02%) |
|  | **BAAI/bge-m3** | **94.00% (±0.03%)** | **97.02% (± 0.02%)** | **97.04%(± 0.02%)** | **97.00%(± 0.02%)** |
|  | dmis-lab/biobert-v1.1 | 92.71% (± 0.03%) | 94.24% (± 0.02%) | 94.33% (± 0.02%) | 94.16% (± 0.02%) |
|  | emilyalsentzer/Bio ClinicalBERT | 89.00% (± 0.01%) | 90.42% (± 0.01%) | 90.52% (± 0.02%) | 90.32% (± 0.01%) |
| Malnutrition risk factors | Alibaba-NLP/gte-large-en-v1.5 | 87.91% (± 0.13%) | 91.45% (± 0.05%) | 92.44% (± 0.02%) | 90.55% (± 0.01%) |
|  | BAAI/bge-largeen-v1.5 | 89.44% (± 0.01%) | 94.78% (± 0.07%) | 95.66% (± 0.05%) | 94.07% (± 0.02%) |
|  | BAAI/bge-m3 | **90.18% (±0.02%)** | **94.86% (± 0.01%)** | **97.29% (± 0.01%)** | **96.99% (± 0.02%)** |
|  | dmis-lab/biobert-v1.1 | 88.47% (± 0.05%) | 95.56% (± 0.02%) | 96.31% (± 0.05%) | 94.82% (± 0.02%) |
|  | emilyalsentzer/Bio ClinicalBERT | 88.81% (± 0.02%) | 95.27% (± 0.02%) | 96.50% (± 0.04%) | 94.05% (± 0.02%) |
| Agitation in dementia | Alibaba-NLP/gte-large-en-v1.5 | 87.20% (± 0.06%) | 87.36% (± 0.05%) | 86.91% (± 0.03%) | 87.81% (± 0.04%) |
|  | BAAI/bge-largeen-v1.5 | 88.19% (± 0.01%) | 89.47% (± 0.01%) | 89.50% (± 0.01%) | 89.44% (± 0.01) |
|  | BAAI/bge-m3 | **90.00% (± 0.03%)** | **90.51% (± 0.01%)** | **90.27% (± 0.03%)** | **90.75% (± 0.06%)** |
|  | dmis-lab/biobert-v1.1 | 89.89% (± 0.03%) | 89.16% (± 0.02%) | 88.50% (± 0.017%) | 89.82% (± 0.02%) |
|  | emilyalsentzer/Bio ClinicalBERT | 89.20% (± 0.03%) | 89.70% (± 0.02%) | 89.50% (± 0.03%) | 89.90% (± 0.03%) |
| Frailty index | LangChain | **94.00% (± 0.03%)** | **97.08% (± 0.02)** | **97.04% (± 0.02%)** | **97.12% (± 0.02%)** |
|  | LlamaIndex | 47.12% (± 0.67%) | 63.93% (± 0.46) | 64.01% (± 0.52%) | 63.85% (± 0.56%) |
| Malnutrition risk factors | LangChain | **90.18% (± 0.02%)** | **97.14% (± 0.04)** | **97.29% (± 0.02%)** | **96.99% (± 0.01%)** |
|  | LlamaIndex | 78.21% (± 0.02%) | 80.46% (± 0.02) | 80.47% (± 0.03%) | 80.45% (± 0.01%) |
| Agitation in dementia | LangChain | **90.00% (± 0.03%)** | **97.08% (± 0.03)** | **97.04% (± 0.02%)** | **97.12% (± 0.02%)** |
|  | LlamaIndex | 77.74% (± 0.02%) | 78.36% (± 0.02) | 77.20% (± 0.03%) | 79.52% (± 0.06%) |
| Frailty index | 1-shot | 91.00% (± 0.02%) | 96.01% (± 0.02%) | 95.96% (± 0.01%) | 96.06% (± 0.01%) |
|  | 2-shot | 91.00% (±0.02%) | 95.09% (± 0.02%) | 95.04%(± 0.02%) | 95.15%(± 0.02%) |
|  | 3-shot | **94.21%** **(± 0.02%)** | **97.08% (± 0.02%)** | **97.04% (± 0.02%)** | **97.12% (± 0.02%)** |
|  | 4-shot | 90.00% (± 0.04%) | 94.25% (± 0.02%) | 94.43% (± 0.02%) | 94.06% (± 0.02%) |
|  | 5-shot | 86.00% (± 0.01%) | 92.46% (± 0.02%) | 92.34% (± 0.02%) | 92.58% (± 0.02%) |
| Malnutrition risk factors | 1-shot | 85.42% (± 0.01%) | 92.02% (± 0.02%) | 94.00% (± 0.05%) | 90.03% (± 0.06%) |
|  | 2-shot | 93.44% (± 0.01%) | 96.58% (± 0.01%) | 97.04% (± 0.02%) | 96.12% (± 0.02%) |
|  | 3-shot | **94.21% (±0.02%)** | **97.20% (± 0.02%)** | **97.29% (± 0.02%)** | **97.12% (± 0.03%)** |
|  | 4-shot | 92.13% (± 0.03%) | 95.55% (± 0.05%) | 95.56% (± 0.03%) | 95.53% (± 0.03%) |
|  | 5-shot | 90.23% (± 0.03%) | 94.45% (± 0.04%) | 94.55% (± 0.04%) | 94.44% (± 0.05%) |
| Agitation in dementia | 1-shot | 85.42% (± 0.13%) | 94.02% (± 0.05%) | 94.11% (± 0.06%) | 93.93% (± 0.06%) |
|  | 2-shot | 93.19% (± 0.03%) | 96.74% (± 0.03%) | 97.04% (± 0.03%) | 96.44% (± 0.03) |
|  | 3-shot | **94.21% (± 0.03%)** | **97.20% (± 0.03%)** | **97.29% (± 0.02%)** | **97.12% (± 0.03%)** |
|  | 4-shot | 92.13% (± 0.03%) | 93.45% (± 0.05%) | 93.56% (± 0.03%) | 93.33% (± 0.03%) |
|  | 5-shot | 90.20% (± 0.03%) | 94.70% (± 0.03%) | 95.50% (± 0.03%) | 93.90% (± 0.05%) |
| Frailty index | K=1 | 71.21% (± 0.028%) | 83.72% (± 0.016%) | 83.64% (± 0.024%) | 83.79% (± 0.019%) |
|  | K=5 | **94.00% (± 0.03%)** | **97.08% (± 0.01%)** | **97.04% (± 0.01%)** | **97.12% (± 0.01%)** |
|  | K=10 | 89.00% (±0.11%) | 93.71% (± 0.05%) | 93.66%(± 0.02%) | 93.76%(± 0.11%) |
|  | K=15 | 86.00% (± 0.014%) | 92.14% (± 0.02%) | 92.16% (± 0.02%) | 92.12% (± 0.02%) |
|  | K=20 | 86.00% (± 0.03%) | 88.53% (± 0.02%) | 91.74% (± 0.026%) | 85.32% (± 0.026%) |
| Malnutrition risk factors | K=1 | 79.21% (± 0.023%) | 83.83% (± 0.015%) | 82.14% (± 0.016%) | 85.52% (± 0.016%) |
|  | K=5 | **90.18% (± 0.02%)** | **97.14% (± 0.01%)** | **97.29% (± 0.01%)** | **96.99% (± 0.01%)** |
|  | K=10 | 88.52% (±0.02%) | 90.21% (± 0.03%) | 90.25% (± 0.03%) | 90.17% (± 0.03%) |
|  | K=15 | 87.52% (± 0.01%) | 90.06% (± 0.03%) | 90.01% (± 0.03%) | 90.12% (± 0.02%) |
|  | K=20 | 86.22% (± 0.02%) | 89.67% (± 0.08%) | 89.47% (± 0.01%) | 89.87% (± 0.01%) |
| Agitation in dementia | K=1 | 80.88% (± 0.03%) | 81.14% (± 0.05%) | 80.87% (± 0.06%) | 81.41% (± 0.06%) |
|  | K=5 | **91.14% (± 0.03%)** | **91.01% (± 0.03%)** | **90.27% (± 0.06%)** | **91.75% (± 0.03)** |
|  | K=10 | 87.55% (± 0.03%) | 87.34% (± 0.05%) | 87.27% (± 0.06%) | 87.41% (± 0.04%) |
|  | K=15 | 85.21% (± 0.03%) | 86.53% (± 0.06%) | 86.54% (± 0.07%) | 86.52% (± 0.04%) |
|  | K=20 | 84.52% (± 0.03%) | 85.66% (± 0.05%) | 85.75% (± 0.04%) | 85.56% (± 0.03%) |
